# Obstructive sleep apnea syndrome and causal relationship with female breast cancer: a mendelian randomization study

**DOI:** 10.18632/aging.102725

**Published:** 2020-02-29

**Authors:** Xiao-Ling Gao, Zhi-Mei Jia, Fang-Fang Zhao, Dong-Dong An, Bei Wang, Er-Jing Cheng, Yan Chen, Jian-Nan Gong, Dai Liu, Ya-Qiong Huang, Jiao-Jiao Yang, Shu-Juan Wang

**Affiliations:** 1 Department of Respiratory and Critical Care Medicine, The Second Hospital of Shanxi Medical University, Taiyuan 030001, P.R. China; 2 The Second Department of Clinical Medicine, Shanxi Medical University, Taiyuan 030000, P.R. China

**Keywords:** mendelian randomization, breast cancer, obstructive sleep apnea syndrome, causal relation

## Abstract

Although observational studies have reported a positive association between obstructive sleep apnea syndrome (OSAS) and breast cancer (BC) risk, causality remains inconclusive. We aim to explore whether OSAS is associated with etiology of BC by conducting a two-sample Mendelian randomization (MR) study in a Chinese population and Asian population from the Breast Cancer Association Consortium (BCAC). We found a detrimental causal effect of OSAS on BC risk in the primary analysis of our samples (IVW OR, 2.47 for BC risk per log-odds increment in OSAS risk, 95% CI = 1.86-3.27; P = 3.6×10^-10^). This was very similar to results of the direct observational case-control study between OSAS and BC risk (OR = 2.80; 95% CI = 2.24-3.50; P =1.4×10^-19^). Replication in the Asian population of the BCAC study also supported our results (IVW OR, 1.33 for BC risk per log-odds increment in OSAS risk, 95% CI = 1.13-1.56; P = 0.0006). Sensitivity analyses confirmed the robustness of our findings. We provide novel evidence that genetically determined higher risk of OSAS has a causal effect on higher risk of BC. Further studies focused on the mechanisms of the relationship between OSAS and breast carcinogenesis are needed.

## INTRODUCTION

Breast cancer (BC) ranks as the most common cancer and the second most common cause of death from cancers in women worldwide [1–4]. According to the report of the Global Burden of Disease (GBD) Study 2017, the estimated annual deaths of BC was 611.6 thousand, and the all-age years of life lost (YLLs) was 16400.7 thousand globally [5]. Sleep-related mechanisms, which might initiate, exacerbate or modulate the phenotypic expression of multiple diseases, have been widely investigated for their relationships with BC [6–8]. However, less investigation has explored the potential detrimental effects of obstructive sleep apnea syndrome (OSAS), which has become a highly prevalent condition throughout the lifespan [9–16].

OSAS, a sleep-related breathing disorder characterized by recurrent cessations of breathing during sleep, could lead to intermittent hypoxia and sleep fragmentation [17]. Chronic and intermittent hypoxia have been shown to play an essential role in the progress of carcinogenesis and tumor progression [18–20]. Many observational studies have implicated the potential detrimental role of OSAS in multiple cancers, although the results were inconsistent [6, 21–24]. The causality of OSAS and BC still remains unknown due to the inherent limitations in observational studies of confounding and reverse causation.

Mendelian randomization (MR), using genetic variants as an instrument variable (IV) for the exposure to estimate causal effects of modifiable risk factors on disease outcomes, could overcome the limitations of the observational studies [25]. It have successfully adopted in a wide spectrum of diseases, including cancers, cardiovascular diseases, diabetes, and so on [16, 26–34]. In current study, we aims to performed a two-sample MR analysis to examine the causal effect of OSAS and etiology of BC.

## RESULTS

### Baseline characteristics of the included samples

As shown in Table 1, two case-control studies were conducted. The first study aimed to replicate the GWAS findings of OSAS, which were mostly identified in European population. Then, we evaluated associations of the positive variants with BC risk in the second case-control study. The distribution of age, body mass index (BMI), and smoking status were comparable between the cases and controls, while BC cases have more family history of cancer, and OSAS (Table 1, P<0.001).

**Table 1:** Characteristics of women included in the mendelian randomization study.

**Variables**	**OSAS**				**BC**		
	**Cases (n=900)**	**Controls (n=1078)**	**P value**		**Cases (n=1200)**	**Controls (n=1200)**	**P value**
Age							
≥50	462 (51.3%)	564 (52.3%)	0.662		685 (57.1%)	641 (53.4%)	0.071
<50	438 (48.7%)	514 (47.7%)			515 (42.9%)	559 (36.6%)	
Body mass index (BMI)	23.97±3.21	23.88±3.17	0.532		23.96±3.20	23.90±3.24	0.648
Family history of cancer							
Yes	113 (12.6%)	108 (10.0%)	0.074		289 (24.1%)	122 (10.2%)	<0.001
No	787 (87.4%)	970 (90.0%)			911 (75.9%)	1078 (89.8%)	
Smoking status							
Smokers	160 (17.8%)	162 (15.0%)	0.099		215 (17.9%)	184 (15.3%)	0.089
Non-Smokers	740 (82.2%)	916 (85.0%)			985 (82.1%)	1016 (84.7%)	
OSAS							
Yes	-	-			289 (24.1%)	122 (10.2%)	<0.001
No	-	-			911 (75.9%)	1078 (89.8%)	

### Replication of OSAS loci and their associations with BC risk in Chinese population

All 23 OSAS risk loci identified by GWASs mostly in European population were presented in Supplementary Table 1. Among them, 13 variants met the standard of minor allele frequency (MAF) ≥ 5% in Chinese Han population and pairwise r2 < 0.8. As shown in Table 2, 5 proxy SNPs, including rs10097555, rs11074782, rs10777373, rs11588454, and rs11897825, were identified to be significantly associated with OSAS risk in Chinese samples (P<0.05). All of these five variants were in agreement with HWE in controls (P > 0.05). As shown in Table 3, we found rs10097555, rs11074782, rs10777373, rs11588454, and rs11897825 were significantly associated with BC risk, after adjusted for age, smoking status, family history of cancer and BMI (P<0.05). Minor alleles of SNP rs11588454 and rs11897825 was associated with increased risk of BC, while those of rs10097555, rs11074782, rs10777373 were associated with decreased risk of BC.

**Table 2:** Replication of the GWAS identified OSAS variants in Chinese population.

	**OSAS cases**	**Controls**	OR (95% CIs) ^*^	**P value**
rs10097555				
AA	621	691	1.00 (Reference)	
AG	267	355	0.84 (0.69-1.01)	0.069
GG	12	32	0.42 (0.22-0.8)	0.009
G vs A			0.80 (0.71-0.90)	0.001
rs11074782				
CC	606	680	1.00 (Reference)	
TC	267	343	0.87 (0.72-1.06)	0.171
TT	27	55	0.55 (0.35-0.88)	0.012
T vs C			0.82 (0.73-0.92)	0.001
rs10777373				
CC	472	513	1.00 (Reference)	
TC	361	446	0.88 (0.73-1.06)	0.179
TT	67	119	0.61 (0.44-0.84)	0.003
T vs C			0.82 (0.73-0.94)	0.003
rs11588454				
TT	479	638	1.00 (Reference)	
TC	360	388	1.24 (1.03-1.49)	0.026
CC	61	52	1.56 (1.06-2.30)	0.023
C vs T			1.25 (1.11-1.40)	0.002
rs11897825				
AA	286	409	1.00 (Reference)	
AG	466	518	1.29 (1.06-1.57)	0.012
GG	148	151	1.40 (1.07-1.84)	0.015
G vs A			1.20 (1.09-1.32)	0.001

* Adjusted for age, smoking status, family history of cancer and BMI.

**Table 3:** Associations of the OSAS variants with BC risk in Chinese population.

	**OSAS cases**	**Controls**	OR (95% CIs) ^*^	**P value**
rs10097555				
AA	827	769	1.00 (Reference)	
AG	351	395	0.83 (0.69-0.98)	0.032
GG	22	36	0.57 (0.33-0.97)	0.038
G vs A			0.82 (0.70-0.95)	0.007
rs11074782				
CC	821	757	1.00 (Reference)	
TC	341	381	0.83 (0.69-0.98)	0.033
TT	38	62	0.57 (0.37-0.85)	0.006
T vs C			0.79 (0.68-0.91)	0.001
rs10777373				
CC	631	571	1.00 (Reference)	
TC	461	498	0.84 (0.71-0.99)	0.041
TT	108	131	0.75 (0.56-0.99)	0.039
T vs C			0.85 (0.75-0.96)	0.009
rs11588454				
TT	651	705	1.00 (Reference)	
TC	461	426	1.17 (0.99-1.39)	0.066
CC	88	69	1.38 (0.99-1.92)	0.056
C vs T			1.18 (1.03-1.34)	0.015
rs11897825				
AA	388	452	1.00 (Reference)	
AG	611	583	1.22 (1.02-1.46)	0.027
GG	201	165	1.42 (1.11-1.82)	0.005
G vs A			1.19 (1.06-1.34)	0.003

* Adjusted for age, smoking status, family history of cancer and BMI.

### MR analyses

The F-statistic for the 5 instrument SNPs were all well above the threshold of F >10 typically recommended for MR analyses. Table 4 presents the summary statistics of the five genetic variants used as instrumental variables in both our sample and Asian population of the BCAC study. Associations of genetically determined risk of OSAS with BC risk using multiple MR methods are shown in Table 5. We found evidence of a detrimental causal effect of OSAS on BC risk in the primary analysis of our samples (IVW OR, 2.47 for BC risk per log-odds increment in OSAS risk, 95% CI = 1.86-3.27; P = 3.6×10^-10^). This was very similar to results of the direct observational case-control study between OSAS and BC risk (OR = 2.80; 95% CI = 2.24-3.50; P =1.4×10^-19^). When we replicated our findings in the Asian population of the BCAC study, we also found the detrimental causal effect (IVW OR, 1.33 for BC risk per log-odds increment in OSAS risk, 95% CI = 1.13-1.56; P = 0.0006). Sensitivity analyses by MBE, penalized IVW, robust IVW, simple median, and weighted median method confirmed the robustness of our findings. For the potential pleiotropy effect, we didn’t find any other associations by searching MR-Base, PhenoScanner database and the GWAS catalog. The intercept from MR-Egger regression didn’t differed from zero (P>0.05). Also, MR-PRESSO analyses revealed that no potential outliers were detected in both our sample and Asian population of the BCAC study.

**Table 4:** Genetic variants used as instrumental variables in summary statistics approach.

**SNPs**	**Effect allele**	**Beta (OSAS)**	**Se (OSAS)**	**Beta (BC)**	**Se (BC)**
**Current study**					
rs10097555	A	0.22	0.06	0.20	0.07
rs11074782	C	0.20	0.06	0.24	0.07
rs10777373	C	0.20	0.06	0.16	0.06
rs11588454	C	0.22	0.06	0.16	0.07
rs11897825	G	0.18	0.05	0.17	0.06
**BCAC study (Asian population)**					
rs10097555	A	0.22	0.06	0.08	0.03
rs11074782	C	0.20	0.06	0.08	0.04
rs10777373	C	0.20	0.06	0.05	0.05
rs11588454	C	0.22	0.06	0.04	0.04
rs11897825	G	0.18	0.05	0.02	0.04

**Table 5:** Genetically predicted associations between OSAS and susceptibility of BC.

**MR methods**	**Current study**			**BCAC study (Asian population)**	
	**OR (95% CI)**	**P value**		**OR (95% CI)**	**P value**
IVW	2.47 (1.86-3.27)	3.6×10^-10^		1.33 (1.13-1.56)	0.0006
MBE	2.31 (1.34-4.01)	2.7×10^-3^		1.42 (1.06-1.91)	0.021
Penalized IVW	2.47 (1.86-3.27)	3.6×10^-10^		1.33 (1.13-1.56)	0.0006
Robust IVW	2.45 (1.89-3.16)	8.4×10^-12^		1.33 (1.19-1.49)	8.9×10^-7^
Simple median	2.48 (1.53-4.03)	2.3×10^-4^		1.28 (1.00-1.64)	0.047
Weighted median	2.43 (1.51-3.91)	2.4×10^-4^		1.34 (1.06-1.70)	0.013

## DISCUSSION

In current study, we applied a two-sample MR approach to comprehensively evaluate the causal relationships of OSAS and etiology of BC in both Chinese samples and Asian population of the BCAC study. The primary MR analyses showed that genetic predisposition to higher risk of OSAS was associated with higher risk of BC. Meanwhile, sensitivity analyses validated the robustness of the primary results. We also didn’t detect any pleiotropy effect of the IV for OSAS using series of methods. To the best of our knowledge, this should be first study which aims to explore the causal relationships between OSAS and risk of BC.

Sleep-related disorders is a series of different medical disorders of the sleep patterns, including dyssomnias, parasomnias, circadian rhythm sleep disorders, and others [35, 36]. Among them, OSAS is the most frequent type of respiratory disturbance [37]. It was estimated that OSAS owned a mean prevalence rate of 22% (range, 9-37%) in men and 17% (range, 4-50%) in women globally [38]. According to the results of a meta-analysis in Asian countries, China and India present the highest prevalence of OSAS [39]. Previous retrospective and prospective observational studies revealed there was a possible association between OSAS and elevated cancer risk, although it was not determined that whether it was a causal relationship [22, 40–43]. Even some studies reported null association or reversed conclusion that cancers and its related therapies caused the occurrence of OSAS [44–46]. Against this background, the implement of MR in the causal inference was much more essential. Recently, a MR analysis evaluated the associations of self-reported chronotype (morning or evening preference), insomnia symptoms, sleep duration, with BC risk using the UK Biobank data [16]. They identified a protective effect of morning preference and suggestive evidence for an adverse effect of increased sleep duration on BC risk [16]. However, OSAS trait was not evaluated due to the complexity of trait measurement.

To make up for this defect, a two-sample MR method was implemented to evaluated the explore the causal relationships between OSAS and risk of BC in current study. Results of both of Chinese samples and the Asian population of the BCAC study revealed that OSAS has a causal effect on higher BC risk. This results supported the previous underpowered and inconsistent studies and provided stronger evidence for the carcinogenesis role of OSAS [23, 46–49]. We included five instrument SNPs, which were reported in previous genome-wide association studies (GWAS) and replicated in our Chinese samples, for the IV construction of OSAS [40, 51].

*In vitro* and *in vivo* experiments have provided many insights into the mechanism of hypoxia in the progress of carcinogenesis and tumor progression of breast cancer. Two main hypoxia markers, CAIX and HIF-1α, have been widely studied and were up-regulated in BC tissues using GEPIA 2 [52]. An HIF-1α/VEGF-A Axis in cytotoxic T cells was involved in the regulation of tumor progression, while loss of HIF-1α in CD8+ T cells could reduce tumor infiltration and tumor cell killing, and altered tumor vascularization [53]. A high amount of adipocytes enhanced BC progression due to the increased expression of HIF-1α [54]. Additionally, higher levels of serum CAIX was significant prognostic biomarkers of shorter PFS for BC, and CAIX could form a transport metabolon with monocarboxylate transporters in human breast cancer cells [55, 56].

This study has several methodological strengths. First, multiple samples to assess the causal effect of OSAS on BC risk. Second, rigor of the IV construction for OSAS. All five variants were GWAS identified and replicated in our samples. The F-statistic for the 5 instrument SNPs were all well above the threshold of F >10 typically recommended for MR analyses. Third, results were confirmed through sensitivity analyses and pleiotropy effect examination. Limitation should be also considered when interpret the results. First should be the limited number of IV variants. In current study, OSAS risk loci identified by GWASs mostly in European population were evaluated first in Chinese population. Only 5 variants replicated to be associated with OSAS in Chinese population. Next step, more GWASs of OSAS conducted in Asian population are needed. Second, shortage of a large-sample cohort limited the authority of evidence. Future large pooling consortia, larger GWAS of OSAS in Asian population and MR studies using individual level data are warranted.

## CONCLUSIONS

In summary, this study provides novel evidence that genetically determined higher risk of OSAS has a causal effect on higher risk of BC. Our results, in combination with previous literature, provide evidence that population-wide screening for OSAS should be recommended as a primary BC prevention strategy. Future research should be best focused on understanding the mechanisms of the relationship between OSAS and breast carcinogenesis.

## MATERIALS AND METHODS

### Study population

In this two-sample MR study, ethical approval was obtained from the Ethical Committee of the Second Hospital of Shanxi Medical University, and all participants signed the informed consent. The determination of OSAS was conducted using an overnight laboratory-based polysomnography (PSG) test, together with the measurement of apnea–hypopnea index (AHI). Then, OSAS was defined as an AHI >5 events/h, and daytime symptoms specific for an OSAS. The diagnosis of BC was determined by histopathological examination. Demographic information was collected from the medical records. During the same period of time, healthy volunteers visiting the same hospital for physical examination were selected as controls. The shared controls was frequency matched by age, ethnicity and body mass index (BMI). Finally, 900 OSAS (1078 controls, 122 OSAS cases were excluded from the controls in this stage) and 1200 BC cases (1200 controls) were included in current study. Ten ml of venous blood was collected from each study subject. Besides, we also applied the summarized iCOGS data of Asian population (6269 BC cases; 6624 controls) from the Breast Cancer Association Consortium (BCAC) to validate our findings [57].

### Variants selection and genotyping

In MR, genetic variants associated with a risk factor are used as IV to infer the true relationship between the risk factor and outcome. Using the GWAS identified loci to construct the IV was the most commonly used method, as the repeatability, accuracy and stability of the results [58]. In current study, we first retrieved the GWAS catalog, and 23 OSAS risk loci were identified, mostly in European population (Supplementary Table 1). Then, the variants were filtered with the standard of minor allele frequency (MAF) ≥ 5% in Chinese Han population and pairwise r2 < 0.8. Thirteen variants were kept. Further, genotyping was performed for these 13 SNPs using the TaqMan allelic discrimination assay on an ABI 7900 system (Applied Biosystems Inc, Foster City, CA, USA). Blind duplicates of 10% randomly selected samples were genotyped to verify the reproducibility of genotype calls; concordance between duplicates was greater than 100% for all pairs.

### Statistical analysis

All statistical analyses were conducted using the R statistical software (version 3.6.1), and all P values are two-tailed, and P < 0.05 was considered significant. The associations of each SNP with OSAS and BC susceptibility were estimated by unconditional logistic regression analyses with odds ratios (ORs) and 95% confidence intervals (CIs).

We selected the random-effect inverse-variance weighted (IVW) method as the primary analyses. Furthermore, model based estimation (MBE), penalized IVW, robust IVW, simple median, and weighted median method were used for sensitivity analyses. We computed F-statistics to quantify the strength of the selected instruments. Besides, three methods were conducted to detect possible pleiotropy. First, we looked up the MR-Base (http://app.mrbase.org/), PhenoScanner database (http://www.phenoscanner.medschl.cam.ac.uk/) and the GWAS catalog (https://www.ebi.ac.uk/gwas/home) for potential associations of all 5 variants in our study with the following BC-related traits and risk factors. Second, we tested whether the intercept from MR-Egger regression differed from zero, which provided evidence of directional pleiotropy. Third, the MR-Pleiotropy Residual Sum and Outlier (MR-PRESSO) was used to identify and correct for potential outliers.

## Supplementary Material

Supplementary Table 1
